# Identification of a novel bacterial receptor that binds tail tubular proteins and mediates phage infection of *Vibrio parahaemolyticus*

**DOI:** 10.1080/22221751.2020.1754134

**Published:** 2020-05-07

**Authors:** Maozhi Hu, Hui Zhang, Dan Gu, Yi Ma, Xiaohui Zhou

**Affiliations:** Department of Pathobiology and Veterinary Science, University of Connecticut, Storrs, CT, USA

**Keywords:** *Vibrio parahaemolyticus*, bacteriophage, tail tubular protein, adsorption, phage receptor

## Abstract

The adsorption of phages to hosts is the first step of phage infection. Studies have shown that tailed phages use tail fibres or spikes to recognize bacterial receptors and mediate adsorption. However, whether other phage tail components can also recognize host receptors is unknown. To identify potential receptors, we screened a transposon mutagenesis library of the marine pathogen *Vibrio parahaemolyticus* and discovered that a *vp0980* mutant (*vp0980* encodes a predicted transmembrane protein) could not be lysed by phage OWB. Complementation of this mutant with wild-type *vp0980 in trans* restored phage-mediated lysis. Phage adsorption and confocal microscopy assays demonstrated that phage OWB had dramatically reduced adsorption to the *vp0980* mutant compared to that to the wild type. Pulldown assays showed that phage tail tubular proteins A and B (TTPA and TTPB) interact with Vp0980, suggesting that Vp0980 is a TTPA and TTPB receptor. Vp0980 lacking the outer membrane region (aa 114–127) could not bind to TTPA and TTPB, resulting in reduced phage adsorption. These results strongly indicated that TTPA and TTPB binding with their receptor Vp0980 mediates phage adsorption and subsequent bacterial lysis. To the best of our knowledge, this study is the first report of a bacterial receptor for phage tail tubular proteins.

## Introduction

Phages are viruses that infect and replicate within bacteria [1]. The DNA or RNA genome of phages is encapsulated by a proteinaceous capsid, and the capsid of many phages is attached to a tail [2]. Based on the morphology of the tail, phages are classified into three families: Myophoviridae, Siphoviridae and Podoviridae, which have long contractile, long noncontractile and short noncontractile tails, respectively [3,4]. Phages have been found wherever bacteria exist, and up to 70% of marine bacteria may be infected by phages [5]. Some phages have a lytic cycle, and these phages can lyse and destroy bacterial cells after replication [6]. Because of lytic activity, phages have become an alternative and ecofriendly biocontrol agent to prevent and control multidrug-resistant bacteria, particularly in aquatic systems [7].

The first step of phage infection is adsorption to the bacterial cells [2,8]. During adsorption, the virion initially binds reversibly to the bacterial cell surface. Such initial or reversible binding occurs through the interaction between phage tail fibres or tail spikes and primary receptors, e.g. carbohydrate chains or proteins. For example, Sf6 binds lipopolysaccharides using tail fibres [9]. Long fibres in T4 phages (myophages) can also recognize outer membrane protein C (OmpC) as the primary receptor to mediate initial adsorption [10]. Tail fibres can also cleave the polysaccharide chain so that phages bind more closely to the bacterial cell surface [11]. However, studies have shown that reversible binding is not obligatory for phage infection, as some phages can still infect even in the absence of tail fibres [12]. Following reversible binding, phages are committed upon irreversible binding with a secondary receptor. One such secondary receptor identified on the gram-positive bacterium *Bacillus subtilis* is YueB [13]. The outer membrane protein NfrA mediates irreversible adsorption of phage N4 to the gram-negative bacterium *Escherichia coli* [14]. Binding with the secondary receptor signals the virion to release its DNA into the bacterial cell. Typical structures of the tail that recognize and bind the bacterial secondary receptors are also known to be tail fibres and tail spikes that are present in the podophage family coliphage T7 and *Salmonella* sp. phage P22, respectively [15,16]. The tail of the podophage T7 is composed of at least four proteins: the connector, tail tubular protein A (TTPA), tail tubular protein B (TTPB) and tail fibre [15,17–20]. It remains to be elucidated whether other tail structures, e.g. TTPA and TTPB, can also recognize host receptors to mediate phage adsorption.

*Vibrio parahaemolyticus* is a halophilic gram-negative bacterium that can cause seafood-associated bacterial gastroenteritis in humans through contaminated raw or undercooked seafood consumption [21–23]. In our previous studies, we isolated the *V. parahaemolyticus* lytic phage vB_VpaS_OWB (abbreviated as phage OWB in this study) [24]. Morphologically, phage OWB belongs to the podophage family, with a short noncontractile tail. Phage OWB can effectively adsorb to the *V. parahaemolyticus* surface and cause cell lysis [24]. However, the underlying mechanisms by which phage OWB adsorbs to *V. parahaemolyticus* and causes bacterial lysis are unknown. In particular, phage ligands and bacterial receptors that are required for adsorption need to be elucidated. In this study, transposon mutagenesis library screening revealed that the predicted *V. parahaemolyticus* transmembrane protein Vp0980 is required for phage OWB adsorption. Further pulldown assays demonstrated that Vp0980 could bind the phage OWB tail tubular proteins A and B (TTPA and TTPB). Lack of such binding lead to reduced phage adsorption and bacterial cell lysis, demonstrating that Vp0980 is the receptor of podophage tail tubular proteins A and B.

## Materials and methods

### Strains and plasmids

All *E. coli* strains and *V. parahaemolyticus* strains were cultured at 37°C in Luria–Bertani (LB) medium supplemented with 1% NaCl. Complementation was conducted by cloning the respective genes into the low-copy vector pMMB207 as described previously [25]. The strains and vectors used in this study are listed in Table S1. The primers used in this study are listed in Table S2. Phage OWB-infected *V. parahaemolyticus* cultures were centrifuged (13,000×*g* at 4°C for 10 min), and the supernatants containing phage OWB were used in this study after filtration with a 0.22 μm filter [26]. Expression of phage OWB genes in DH5α was performed using the expression plasmid pGEX-4T-1 as described previously [27]. Expression of His- or GST-tagged proteins was induced by 1 mM isopropyl β-D-1-thiogalactopyranoside (IPTG). An LPS mutant was constructed by using the suicide vector pDM4 to knock out the entire operon (*vp0190-vp0214*) for lipopolysaccharide biosynthesis as described previously [25]. Briefly, the upstream of vp0190 (∼500 bp) and the downstream of vp0214 (∼500 bp) was amplified. The two amplified fragments were ligated and cloned to pDM4 vector. After two crossovers and sucrose selection, the mutant with deletion of vp0190-vp0214 was isolated and confirmed by PCR.

### Phage genome sequencing

DNA of phage OWB was extracted as previously described [26]. Briefly, after polyethylene glycol (PEG) precipitation, the phage pellet was resuspended in sodium chloride magnesium sulfate (SM) buffer. Proteinase K (200 μg) and SDS (0.5% final concentration) were added, and the mixture was incubated at 56°C overnight. Proteins were removed by phenol:chloroform:isoamyl alcohol (25:24:1) precipitation, and the nucleic acid was precipitated with alcohol. Finally, the pellets were resuspended in TE buffer (10 mM Tris, pH 8.0, 1 mM EDTA). Whole-genome sequencing was performed using the Illumina HiSeq platform, and the sequence was deposited in GenBank under the accession number MN974282.

### Transposon mutagenesis of *V. parahaemolyticus*

A transposon mutant library of ATCC17802 was constructed with the conjugal helper plasmid pEVS104 and Mini-Tn5 delivery plasmid pEVS170 as described previously [28]. The mutants were selected on LB agar plates supplemented with carbenicillin (50 μg/ml) and erythromycin (10 μg/ml). Approximately 5,000 mutants were screened in the phage drop assay, as described below, to identify those that could not be lysed by phage OWB.

### Construction of plasmids

The coding sequences for OWB027, OWB028, OWB030, OWB031 and OWB035 were PCR amplified from phage OWB using the primer pairs OWB027_FwBamH I/OWB027_ReEcoR I, OWB028_FwBamH I/OWB028_ReEcoR I, OWB030_FwBamH I/OWB030_ReEcoR I, OWB031_FwBamH I/OWB031_ReEcoR I and OWB035_FwBamH I/OWB035_ReEcoR I, respectively. The resulting PCR products were digested with *BamH* I and *EcoR* I and inserted into the plasmid pGEX that was predigested with *BamH* I and *EcoR* I, resulting in the plasmids pGEX-OWB027, pGEX-OWB028, pGEX-OWB030, pGEX-OWB031 and pGEX-OWB035, respectively (Table S1). These plasmids were used to express GST-tagged OWB027, OWB028, OWB030, OWB031 and OWB035. The *vp0980* gene was amplified using the primer pair pmmbvp0980_1F/pmmbvp0980_2R. A 6xHis tag was added at the C-terminus of the encoded protein. The PCR product was inserted into *Hind* III/*Xba* I double-digested pMMB207 [25], resulting in the plasmid pMMB207-vp0980 (Table S1). This plasmid was used in complementation and pulldown assays. Similarly, *vp0879* was amplified with pmmbvp0879_1F/pmmbvp0879_2R and inserted into pMMB207, resulting in the plasmid pMMB207-vp0879 (Table S1). To express *vp0980* lacking its transmembrane or outer regions, the up- and downstream regions flanking amino acids 91–113, 114–127 and 128–150 of Vp0980 were amplified from *V. parahaemolyticus* using the primer pairs pmmbvp0980_1F/pmmbvp0980_91_1R and pmmbvp0980_91_2F/pmmbvp0980_2R, pmmbvp0980_1F/pmmbvp0980_114_1R and pmmbvp0980_114_2F/pmmbvp0980_2R, and pmmbvp0980_1F/pmmbvp0980_128_1R and pmmbvp0980_128_2F/pmmbvp0980_2R (Table S2), respectively. The resulting upstream and downstream products were inserted into *Hind* III/*Xba* I double-digested pMMB207, resulting in the plasmids pMMB207-vp0980Δ91-113, pMMB207-vp0980Δ114-127 and pMMB207-vp0980Δ128-150 (Table S1), respectively. These plasmids were used to complement Δ*vp0980*. To express *vp0879* with a point mutation, the primers pmmbvp0879_1F/pmmbvp0879_K54A_1R and pmmbvp0879_K54A_2F/pmmbvp0879_2R (Table S2) were used to amplify two PCR products that were cloned into pMMB207, resulting in the plasmid pMMB207-vp0879K54A (Table S1).

### Phage drop assay

A phage drop assay was performed as previously described [26]. Briefly, freshly cultured *V. parahaemolyticus* strains were dropped on LB plates (approximately 10^4^ CFU/drop). After the bacterial culture dried, phage OWB was dropped on top of the dried bacterial lawn. After 6 h of incubation at 37°C, clear zones were recorded to reflect the bacterial cell lysis. Each experiment was repeated three times, and representative images are shown.

### Confocal microscopy

For visualization of phage attachment, phages were stained with the fluorescent dye SYBR Green as previously described [29]. Briefly, the phages were stained with SYBR Green for 15 min at 4°C in the dark. Subsequently, the mixture was precipitated by PEG/NaCl for 1 h on ice in the dark. After centrifugation at 13,000×*g* at 4°C for 20 min, the pellet containing phages was resuspended in SM buffer. All *V. parahaemolyticus* strains were transformed with the plasmid pVSV208, which constitutively expresses red fluorescent protein (RFP) [30]. Exponentially growing *V. parahaemolyticus* strains (red) were infected with the SYBR Green-labelled phages (green) at an MOI of 10 for 30 min. Subsequently, infected bacteria were centrifuged, and the bacteria in the pellet were resuspended in phosphate-buffered saline (PBS) and visualized using a confocal microscope. Representative images of at least three experiments are shown. To determine if GST-TTPA and GST-TTPB bind the whole cells of *V. parahaemolyticus*, a bacterial culture was resuspended in 50 µl PBS to reach a concentration of 10^6^ CFU/ml and incubated with 10 μl of the purified recombinant protein GST-TTPA, GST-TTPB or GST (0.5 mg/ml) for 1 h. After extensive washing with PBS, bacterial cells were incubated sequentially with a mouse primary anti-GST antibody and Alexa Fluor 594-conjugated secondary anti-mouse IgG before visualization with a confocal microscope.

### Phage adsorption assay

Adsorption was analyzed as previously described [26]. Briefly, to monitor phage adsorption, phage OWB was mixed with a fresh *V. parahaemolyticus* culture to reach an MOI of 0.01. After incubation at 37 °C for 5, 10, 20, 30 min or 60 min, the phage-bacteria mixture was centrifuged at 12,000 rpm for 10 min. The free phage titre (pfu) in the supernatant was determined. The percent adsorption was determined as follows: percent adsorption = (pfu_added_-pfu_supernatant_)/pfu_added_, and the average data of at least three experiments are shown for each time point. To determine if TTPA and TTPB would block phage adsorption, we incubated wild type *V. parahaemolyticus* with GST-TTPA or GST-TTPB or GST at the concentration of 0.1 mg/ml for 1 h, and subsequently phage adsorption assay was performed as described above.

### Pulldown and western blot assays

The GST-fusion proteins OWB027, OWB028, OWB030, OWB031 and OWB035 from cellular lysates were bound on glutathione agarose beads. After washing with PBS, the membrane protein 6xHis-Vp0980 (solubilized in PBS containing 1% Triton X-100) was then added to the preloaded beads. After additional washing with PBS, the bound proteins were eluted using a buffer containing reduced glutathione. The elution was used for western blotting with anti-GST and anti-His monoclonal antibodies. A similar pull down experiment was also carried out using 6xHis-Vp0980^Δ114–127^. To determine if GST-TTPA and GST-TTPB bind whole cells of *V. parahaemolyticus*, a bacterial culture was resuspended in 50 μl of PBS to reach a concentration of 10^6^ CFU/ml and incubated with 10 μl of the purified recombinant protein GST-TTPA, GST-TTPB or GST (0.5 mg/ml) for 1 h. After extensive washing with PBS, bacterial cells were lysed, and the cell lysate was blotted with an anti-GST antibody. An anti-RNA polymerase (RNAP) antibody was used to indicate that equal bacterial protein was loaded across different samples. To determine whether phage fibre protein (OWB035) binds to whole cells, we incubated wild-type (WT) or LPS mutant with GST-OWB035 or GST. A western blot using anti-GST and anti-RNAP antibodies was performed similarly as described above.

### Bacterial cell growth assay

A bacterial culture (1 ml) at a concentration of approximately 10^4^ CFU/ml (with or without preincubation with GST-TTPA or GST-TTPB at the concentration of 0.1 mg/ml for 1 h) was mixed with phage OWB at the MOI of 10. CFU were determined at different time points. To determine the effect of LPS on phage OWB infection, a bacterial culture of both wild type and LPS mutant (1 ml) at the concentration of approximately 10^4^ CFU/ml was mixed with phage OWB at the MOI of 10. CFU were determined at different time points.

## Results

### Identification of Vp0980 that is required for phage infection of *V. parahaemolyticus*

**Figure 1.**
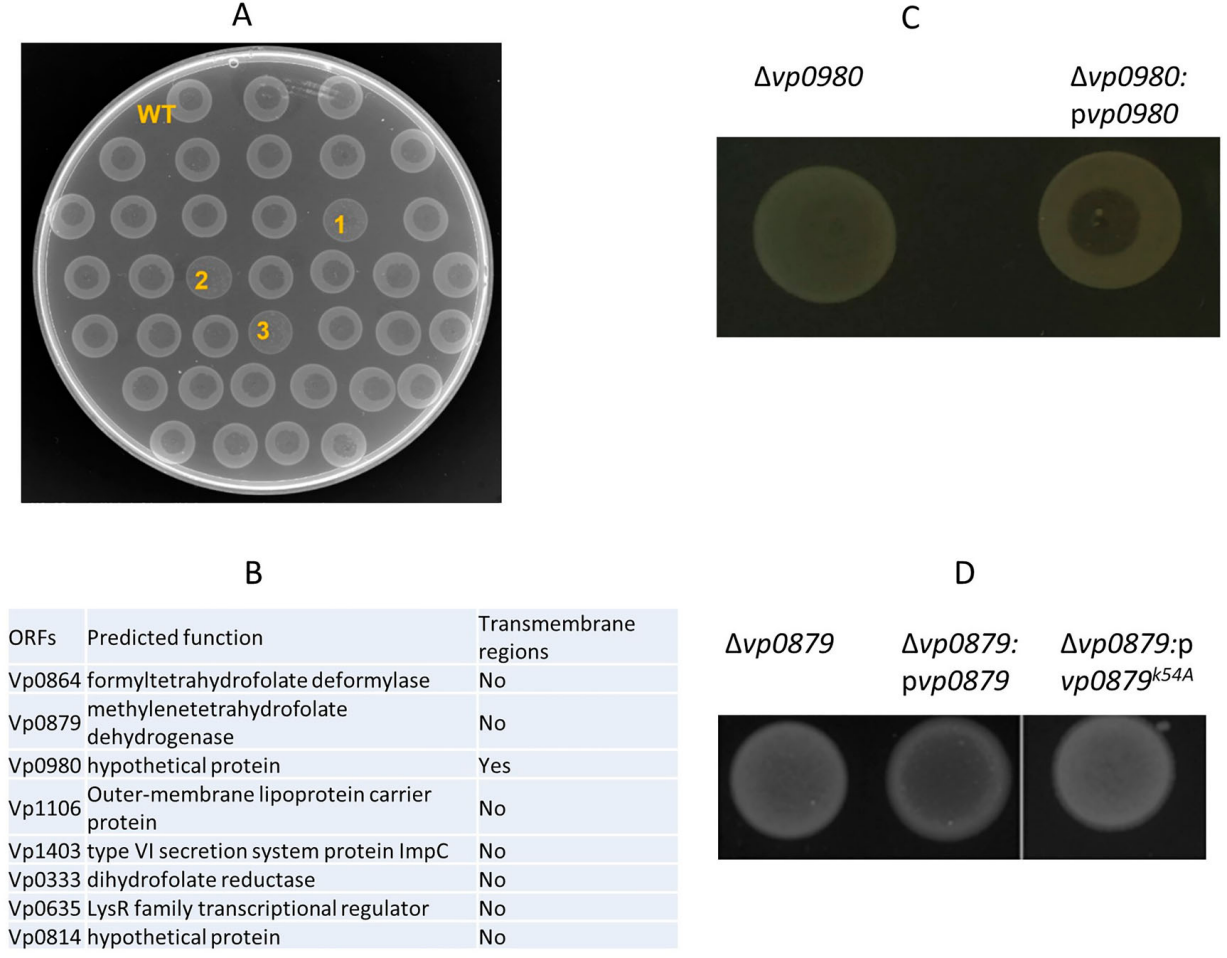
Identification of genes required for phage OWB-mediated lysis of *V. parahaemolyticus.* **(**A) Each mutant in the transposon mutagenesis library was drop plated on LB agar. After drying, a drop of phage (2 µl) was placed on top of the dried bacterial lawn. An example of a phage drop assay used to screen transposon mutants that could not be lysed by phage OWB is shown in A. The top row indicates the WT *V. parahaemolyticus* that can be lysed by phage OWB, showing a clear lysis zone at the center of the bacterial lawn. The majority of mutants could be lysed by phage OWB. The mutants labeled with “1”, “2” and “3” are examples that were not lysed by phage OWB. (B) After screening approximately 5,000 mutants in the transposon mutagenesis library, 8 mutants with transposon insertions were identified to be resistant to phage-mediated lysis. The genes required for phage-mediated lysis are shown, with predicted function and presence or absence of transmembrane regions. (C) Δ*vp0980* complementation with WT *vp0980* restored phage-mediated lysis in the drop assay. (D) Δ*vp0879* complementation with WT *vp0879* but not *vp0879*^K54A^ restored phage-mediated lysis in the drop assay. Representative images from three experiment replications are shown.

Our previous studies have shown that phage OWB causes efficient cell lysis in the *V. parahaemolyticus* strain RIMD2210633 when the polar flagellum is knocked out [24]. The *V. parahaemolyticus* strain 17802 is naturally susceptible to phage OWB infection and can be lysed by phage OWB. Therefore, in this study, we used the strain 17802 as the WT host to identify potential receptors for phage OWB. We first screened a *V. parahaemolyticus* 17802 transposon mutagenesis library to identify mutants that could not be lysed by phage OWB. A clear lysis zone was present at the centre of the WT strain in the phage drop assay, in which phage OWB was placed at the centre of the bacterial lawn (Figure 1A, top row). Examples of mutants that were not lysed by phage OWB are labelled as 1, 2 and 3 in Figure 1A. A total of approximately 5,000 mutants were screened, and we found that 48 mutants could not be lysed by phage OWB. The transposon insertion sites for these 48 mutants were determined by sequencing. The results showed that transposon insertion sites were located in eight open reading frames (ORFs) (Figure 1B). These ORFs encode formyltetrahydrofolate deformylase (Vp0864), methylenetetrahydrofolate dehydrogenase (Vp0879), outer membrane lipoprotein carrier protein (Vp1106), type VI secretion system protein ImpC (Vp1403), dihydrofolate reductase (Vp0333), LysR transcriptional regulator (Vp0635), hypothetical protein Vp0814, and hypothetical protein Vp0980, indicating that these ORFs are involved in any steps of phage infection. One of the mutants that we were most interested in harboured a transposon insertion in the ORF *Vp0980*, as it encodes a predicted transmembrane protein and could potentially serve as receptor for phage adsorption. To exclude the possibility of a polar effect due to transposon insertion, we performed complementation, and the results showed that complementation of the *Vp0980* mutant with WT *Vp0980 in trans* on a plasmid restored cell lysis in the phage drop assay (Figure 1C), indicating that *Vp0980* is essential for phage infection.

### Vp0980 mutation affects phage adsorption to *V. parahaemolyticus*

**Figure 2.**
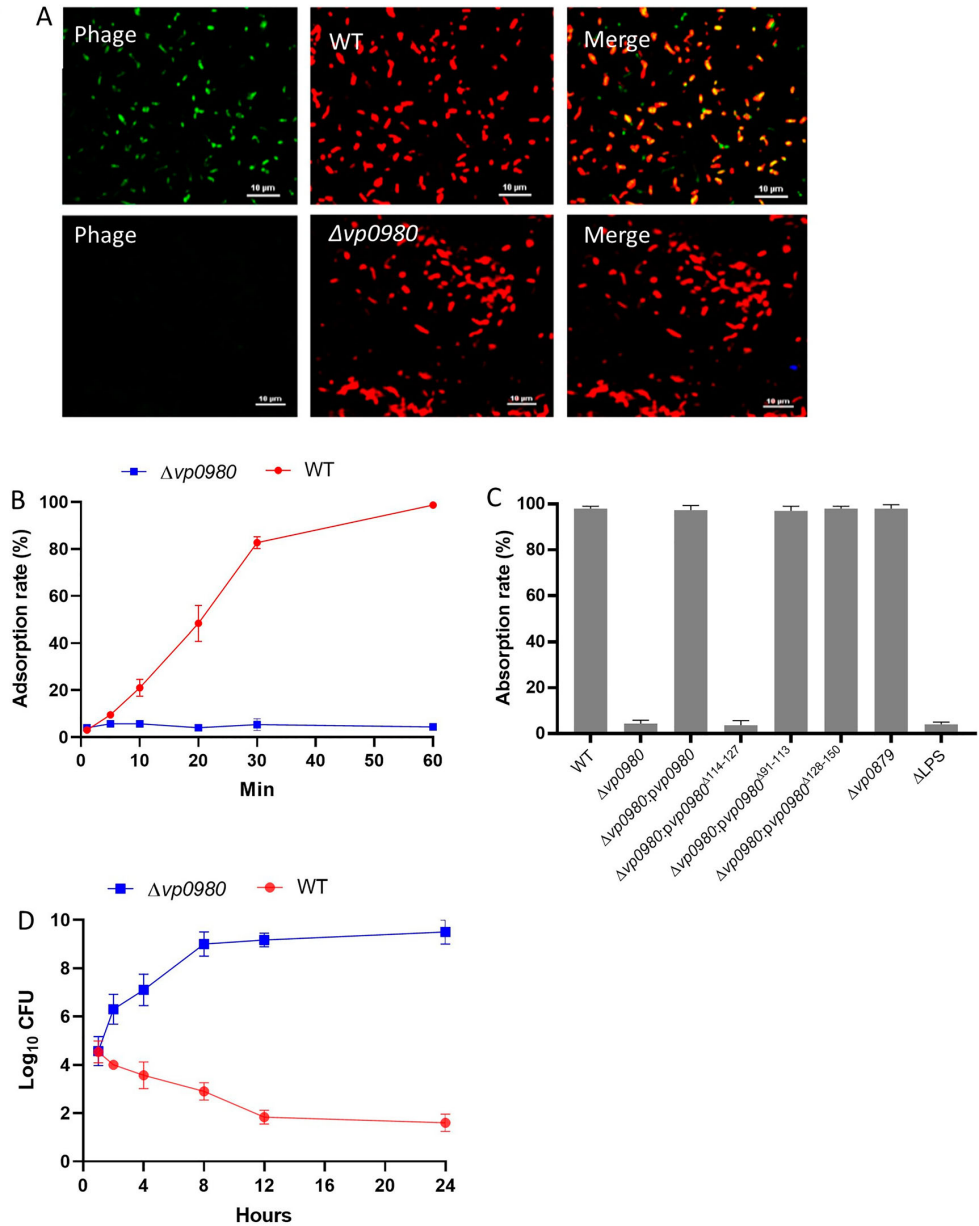
Vp0980 is responsible for phage adsorption onto *V. parahaemolyticus*. RFP-labeled WT (upper panel) or ∆*vp0980* (lower panel) *V. parahaemolyticus* was mixed with SYBR Green-labeled phage for 30 min and subjected to confocal microscopy analysis. Yellow in the merged panel indicates a close association between the phage and bacteria (A). Adsorption quantification for the wild type and Δ*vp0980* (B). Free phage in the supernatant was titrated after the wild type or Δ*vp0980* were mixed with phage for 5, 10, 20, 30 or 60 min, and the phage adsorption percent was calculated as described in the Materials and Methods (B). Adsorption quantification for the WT, mutant and complemented strains as indicated after mixing with phage for 60 min (C). Growth curve of the wild type or Δ*vp0980* in the presence of phage (D). Y-axis represents Log_10_ CFU/ml and X-axis represent hours after growth (D).

We next determined whether mutation of Vp0980 affects phage OWB adsorption to *V. parahaemolyticus*. Confocal microscopy was performed using SYBR Green-labelled phage and RFP-labelled bacteria. The results showed that the majority of the WT bacterial cells had intense green fluorescence after infection (Figure 2A, upper panel). In contrast, the *Vp0980* mutant did not show green fluorescence after infection with SYBR Green-labelled phages (Figure 2A, lower panel). We further quantified the phage adsorption rate in both WT and mutant cells by titrating the supernatant after infection. After 60 min of incubation, over 99.9% of the phages adsorbed to the WT strain (Figure 2B). In contrast, only ∼3–4% of the phages adsorbed to Δ*vp0980* (Figure 2B). Complementation of Δ*vp0980* with WT *vp0980* restored the adsorption rate to >99.9% (Figure 2C). Phage OWB inhibited the growth of the WT strain but not Δ*vp0980* (Figure 2D). These results indicated that phage adsorption to Δ*vp0980* and subsequent lysis were dramatically reduced compared to those with its parental WT strain. Thus, Vp0980 could serve as a potential receptor for phage OWB to adsorb and infect *V. parahaemolyticus*. Interestingly, mutation of *vp0879*, which encodes methylenetetrahydrofolate dehydrogenase (Figure 1B), did not affect phage adsorption (Figure 2C) but did abolish phage-mediated lysis (Figure 1D), indicating that Vp0879 affects phage infection at steps after adsorption. The residue K54 is the predicted catalytic site of methylenetetrahydrofolate dehydrogenase [31]. Thus, we complemented Δ*vp0879* with either WT *vp0879* or *vp0879*^K54A^ (*vp0879* with a K54A point mutation). The phage drop assay showed phage-mediated lysis in Δ*vp0879*:p*vp0879* but not in Δ*vp0879*:p*vp0879*^K54A^ (Figure 1D), indicating that Vp0879 methylenetetrahydrofolate dehydrogenase enzymatic activity is important for phage infection processes after adsorption.

### Genome sequencing of phage OWB

**Figure 3.**
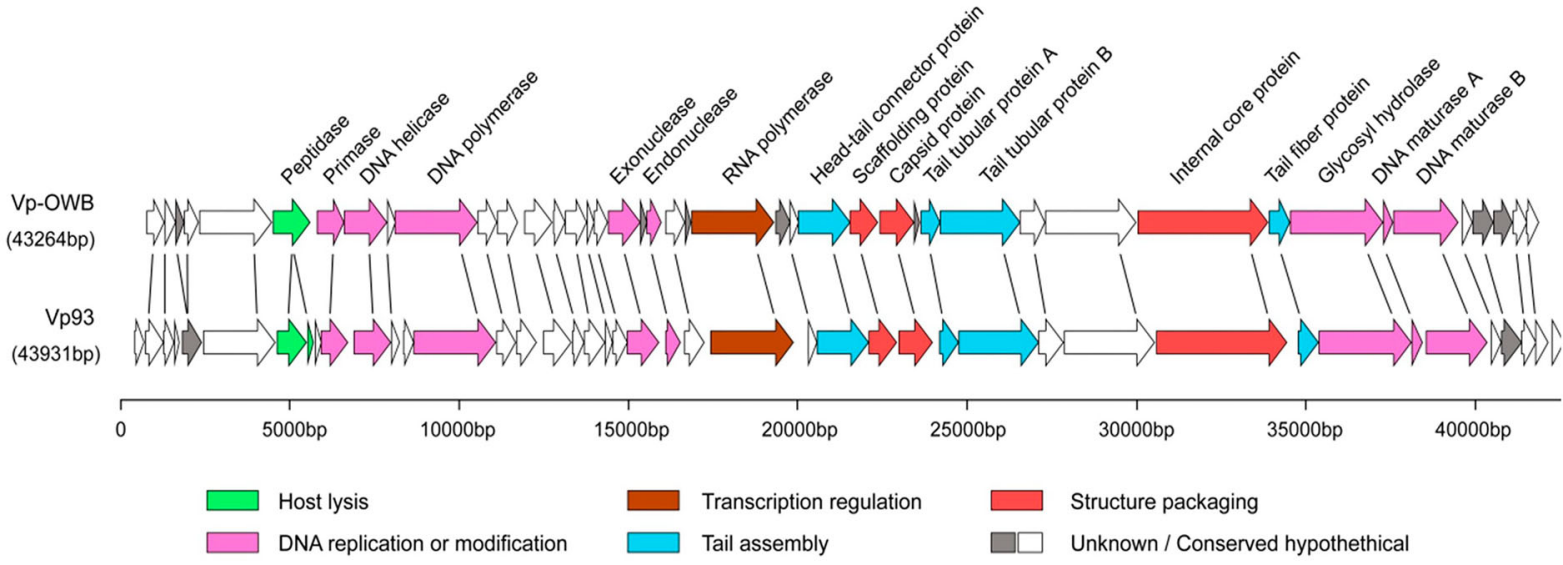
Genome sequencing of phage OWB. Genomic map of phage OWB. Predicted ORFs in the genome of phage OWB (upper panel) and phage VP93 (lower panel) are indicated as arrows. Genes encoding hypothetical proteins are indicated as open arrows, while genes encoding annotated proteins in 6 predicted functional categories are indicated as colored arrows. Each specific function is labeled under the genome map. The scale units are in base pairs.

To determine the specific structure/proteins of phage OWB that bind Vp0980, we first performed whole-genome sequencing for phage OWB. Sequence analysis revealed that the phage OWB genome is 43,264 bp of double-stranded DNA with 43 ORFs and a G + C content of 46.49% (Table S3). Comparative analysis showed that 88.3% (38/43) of the phage OWB ORFs share homology with the *V. parahaemolyticus* phage VP93 that was isolated from the Pacific Ocean off the coast of Chile [32] (Figure 3). Genes for rRNA, tRNA, antibiotic resistance, lysogeny and virulence were not detected in the phage OWB genome. The phage OWB genome has a typical modular structure that includes modules for DNA replication and modification, structure and packaging, tail assembly, host lysis, additional functions and hypothetical proteins. The DNA packaging module of phage OWB is composed of the scaffolding protein (OWB027), capsid protein (OWB028) and internal core protein (OWB034). The DNA replication or modification module is composed of DNA primase, helicase, polymerase, exonuclease, endonuclease, hydrolase and maturase. A host lysis-related protein peptidase (OWB006) was detected, consistent with the observation that phage OWB can lyse host cells. Genes that share homology to known small terminase subunit or integrase were not detected. Genes that encode typical tail structures for podophages include OWB026 (connector), OWB030 (tail tubular protein A, TTPA), OWB031 (tail tubular protein B, TTPB) and OWB035 (tail fibre) (Figure 3 and Table S3).

### Identification of phage proteins that bind Vp0980

**Figure 4.**
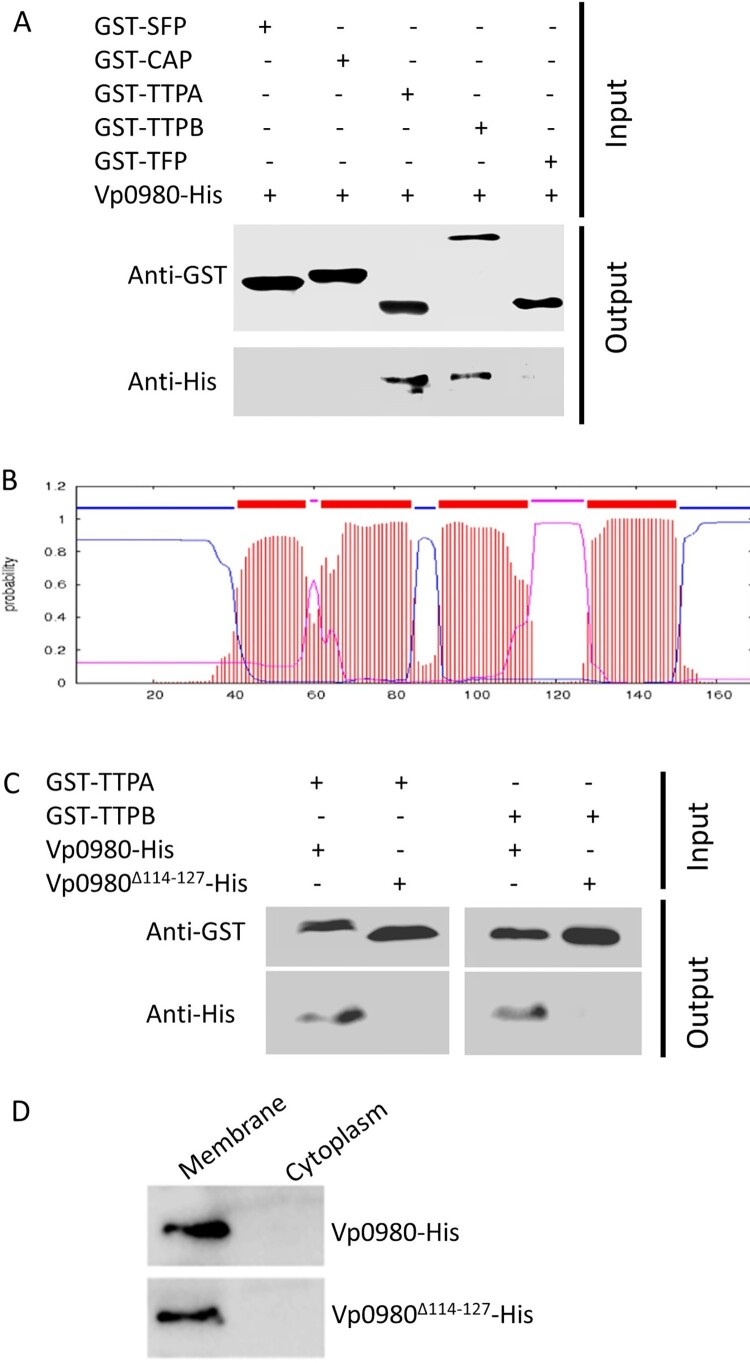
Vp0980 interacts with TTPA and TTPB. (A) GST-fusion of scaffold protein (GST-SFP), capsid protein (GST-CAP), tail tubular protein A (GST-TTPA), tail tubular protein B (GST-TTPB) and tail fiber protein (GST-TFP) were bound on glutathione agarose beads. After washing, 6xHis-tagged Vp0980 was added to the glutathione beads preloaded with each phage protein (input). After additional washing, the bound proteins were eluted for western blotting using anti-GST (upper panel) or anti-His (lower panel) monoclonal antibodies (output). (B) Bioinformatics analysis of Vp0980 with the TMHMM Server revealed four transmembrane regions (extracellular regions are shown as pink, and intracellular regions are shown as blue). (C) A pulldown assay was performed using GST-TTPA or GST-TTPB and 6xHis-tagged Vp0980 or *Vp0980*^Δ114-127^ as the input. Western blotting was performed using anti-GST or anti-His antibodies for elution (output). (D) Localization of Vp0980 and Vp0980^Δ114-127^. Δ*vp0980*:p*vp0980* (upper panel) or Δ*vp0980*:p*vp0980*^Δ114-127^ (lower panel) were cultured, and the bacterial pellet was sonicated. Following centrifugation, the supernatant was ultracentrifuged to separate cytoplasmic proteins (supernatant) and membrane proteins (pellet). Both cytoplasmic (right lane) and membrane proteins (left lane) were subjected to western blotting using an anti-His antibody.

The structures that podophages use primarily to bind receptors (e.g. LPS, teichoic acids, pili, and outer membrane proteins) and mediate adsorption to the host are tail fibres or tail spikes [9,33–39]. We attempted to determine whether other structures of phages are involved in host receptor recognition and binding. Genome sequencing indicated that phage OWB had ORFs encoding two tail tubular proteins (TTPA, OWB030 and TTPB, OWB031). TTPA forms the attachment site for the side fibres, while TTPB (also called a nozzle) serves as an adaptor for mounting additional functions [15,40,41]. Although TTPA and TTPB have been implicated as phage adhesins for adsorption to the host [42,43], it has not been shown whether they bind specific receptors to mediate the adsorption. We expressed GST-tagged TTPA and TTPB and performed pulldown assays with His-tagged Vp0980. The results showed that His-tagged Vp0980 was present in the elution of glutathione agarose preloaded with GST-TTPA or GST-TTPB (Figure 4A), indicating that Vp0980 interacts with TTPA and TTPB. In contrast, His-tagged Vp0980 was not captured by GST-SFP (OWB027, encoding the putative scaffolding protein), GST-CAP (OWB028, encoding the capsid protein) or GST-TFP (OWB035, encoding the tail fibre protein) (Figure 4A), indicating that the tail fibre, capsid and scaffolding proteins do not bind Vp0980. These results demonstrated that Vp0980 specifically binds TTPA and TTPB and could potentially serve as the receptor for TTPA and TTPB to mediate phage adsorption to *V. parahaemolyticus*.

### The outer membrane region of Vp0980 is essential for TTPA and TTPB binding

It is predicted that Vp0980 harbours four transmembrane regions (aa 41–58, aa 62–84, aa 91–113 and aa 128–150), two regions that are inside of the membrane (aa 85–90 and aa 151–169) and two regions that are outside of the membrane (aa 59–61 and aa 114–127) (Figure 4B). We were particularly interested in the regions that are outside of the membrane, as they are the most likely to serve as the binding sites for TTPA and TTPB. We hypothesized that aa 114–127 are the most likely to be the binding site, as the other predicted exterior region (aa 59–61) contains only 3 amino acids and thus is less likely to have a sufficient exterior region for binding. Therefore, we determined the role of aa 114–127 in TTPA and TTPB binding. A pulldown assay showed that His-tagged full-length Vp0980 was present in the elution of glutathione agarose preloaded with GST-TTPA (Figure 4B, the first lane) or GST-TTPB (Figure 4B, the third lane). In contrast, His-tagged Vp0980^Δ114–127^ (Vp0980 lacking aa 114–127) was not present in the elution of glutathione agarose preloaded with GST-TTPA (Figure 4B, the second lane) or GST-TTPB (Figure 4B, the fourth lane). To exclude the possibility that deletion of aa 114–127 may alter the localization of Vp0980, we fractioned the bacterial cells into membrane and cytoplasmic portions, and the results showed that both His-tagged Vp0980 and His-tagged Vp0980^Δ114–127^ were localized on the membrane (Figure 4D), indicating that the deletion of aa 114–127 did not alter the overall membrane localization of Vp0980. Overall, these results strongly indicated that aa 114–127 is the region that directly or indirectly participates in the interaction of Vp0980 with TTPA and TTPB.

### TTPA and TTPB bind bacterial whole cells in a Vp0980-dependent manner

**Figure 5.**
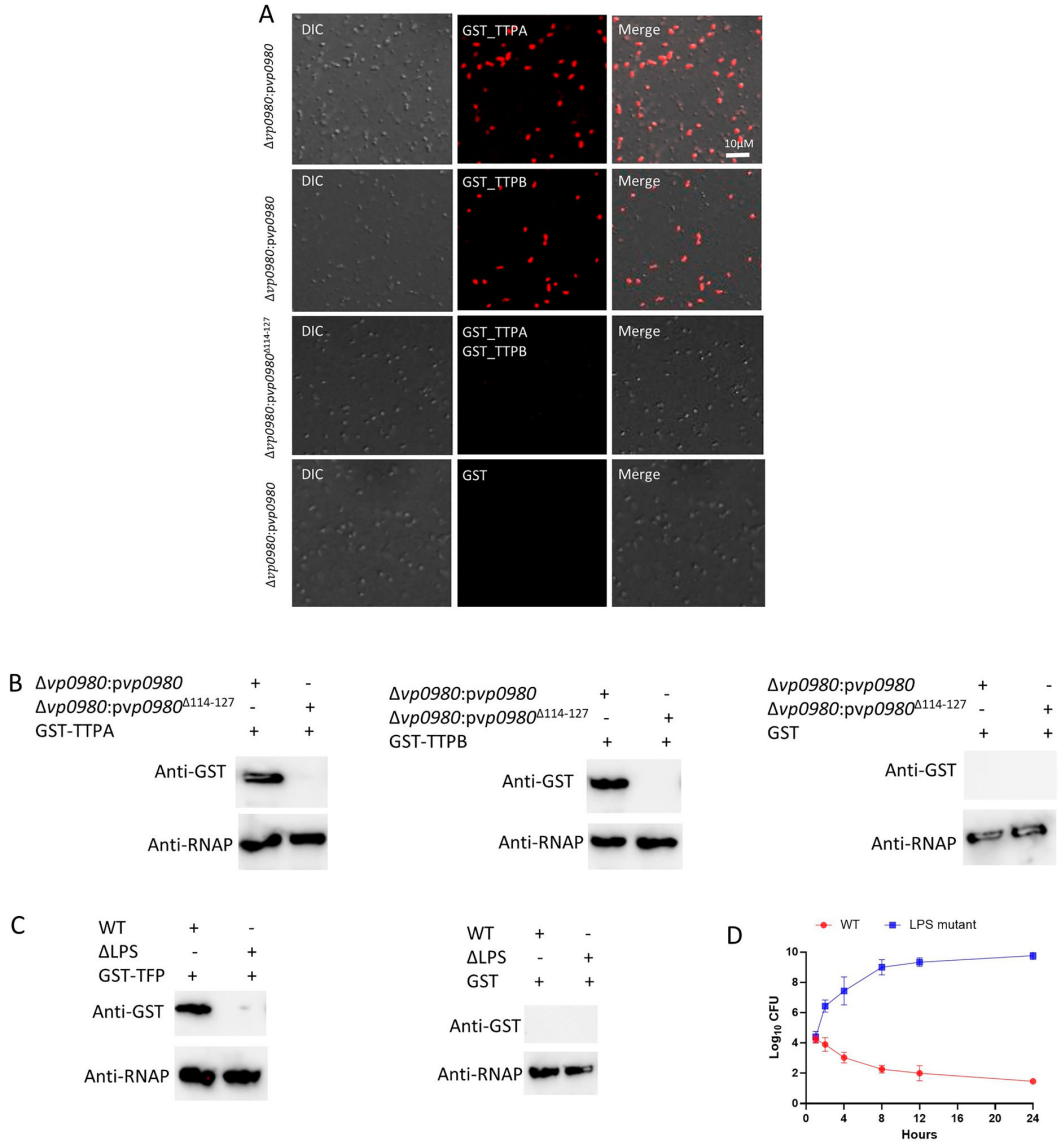
TTPA and TTPB bind whole *V. parahaemolyticus* cells. (A) Whole Δ*vp0980*:p*vp0980* cells were incubated with recombinant GST-TTPA (first panel), GST-TTPB (second panel) or GST alone (fourth panel). Δ*vp0980*:p*vp0980*^Δ114-127^ was incubated with recombinant GST-TTPA and GST-TTPB (third panel). An Alexa Fluor 594-conjugated antibody was used to indicate the presence TTPA and TTPB on the bacterial surface. (B) Whole Δ*vp0980*:p*vp0980* or Δ*vp0980*:p*vp0980*^Δ114-127^ cells were incubated with recombinant GST-TTPA, GST-TTPB or GST alone. After extensive washing, bacterial cells were lysed, and the lysate was subjected to western blotting using anti-GST or anti-RNAP antibodies. (C) Whole WT or ΔLPS cells were incubated with recombinant GST-TFP (predicted tail fiber protein). After extensive washing, the bacterial cells were lysed, and the lysate was subjected to western blotting using anti-GST or anti-RNAP antibodies. RNAP was used to indicate that equal amount of bacterial cells were used for protein incubation. D. Growth curve of the wild type and ΔLPS in the presence of phage. Y-axis represents Log_10_ CFU/ml and X-axis represent hours after growth (D).

To determine if TTPA and TTPB bind Vp0980 in the context of whole bacterial cells, we performed confocal microscopy by incubating the whole cells of Δ*vp0980*:p*vp0980* or Δ*vp0980*:p*vp0980*^Δ114–127^ with recombinant GST-tagged TTPA or TTPB, followed by incubation with a primary mouse anti-GST antibody and secondary Alexa Fluor 594-conjugated anti-mouse IgG. The results showed that both GST-TTPA and GST-TTPB bound the whole cells of Δ*vp0980*:p*vp0980* (Figure 5A, first and second panels). In contrast, GST-TTPA and GST-TTPB did not bind Δ*vp0980*:p*vp0980*^Δ114–127^ (Figure 5A, third panel). As a control, GST alone did not bind Δ*vp0980*:p*vp0980* (Figure 5A, fourth panel). We further performed a whole-cell pulldown by incubating Δ*vp0980*:p*vp0980* or Δ*vp0980*:p*vp0980*^Δ114–127^ with recombinant GST-tagged TTPA or TTPB, followed by extensive washing and western blot analysis of the cell lysate using anti-GST and anti-bacterial RNA polymerase (RNAP) antibodies. RNAP was used to indicate that equal amount of bacterial cells was used across different samples. The results showed that GST-TTPA and GST-TTPB bound the whole cells of Δ*vp0980*:p*vp0980* (Figure 5B) but not the whole cells of Δ*vp0980*:p*vp0980*^Δ114–127^ (Figure 5B). As a control, GST alone did not bind the whole cells of either Δ*vp0980*:p*vp0980* or Δ*vp0980*:p*vp0980*^Δ114–127^ (Figure 5B). These results demonstrated that TTPA and TTPB bind the extracellular region of Vp0980 in the context of whole bacterial cells. As the phage fibre protein typically recognizes bacterial LPS to mediate adsorption, we determined whether OWB035, encoding a putative fibre protein, binds LPS of whole bacterial cells. The results showed that GST-TFP (tail fibre protein) binds to whole cells of the WT strain but not the LPS mutant (Figure 5C). GST alone did not bind the whole cells of either the WT strain or LPS mutant (Figure 5C). Further analysis showed that phage OWB adsorption to LPS mutant was dramatically reduced (Figure 2C). In addition, phage OWB inhibited the growth of the WT strain but not the LPS mutant, indicating that LPS is also important for phage adsorption and infection (Figure 5D). These results indicated that LPS and Vp0980 can be recognized by different phage proteins to mediate phage infection.

### Binding of TTPA and TTPB with Vp0980 is essential for phage adsorption and lysis of bacterial cells

**Figure 6.**
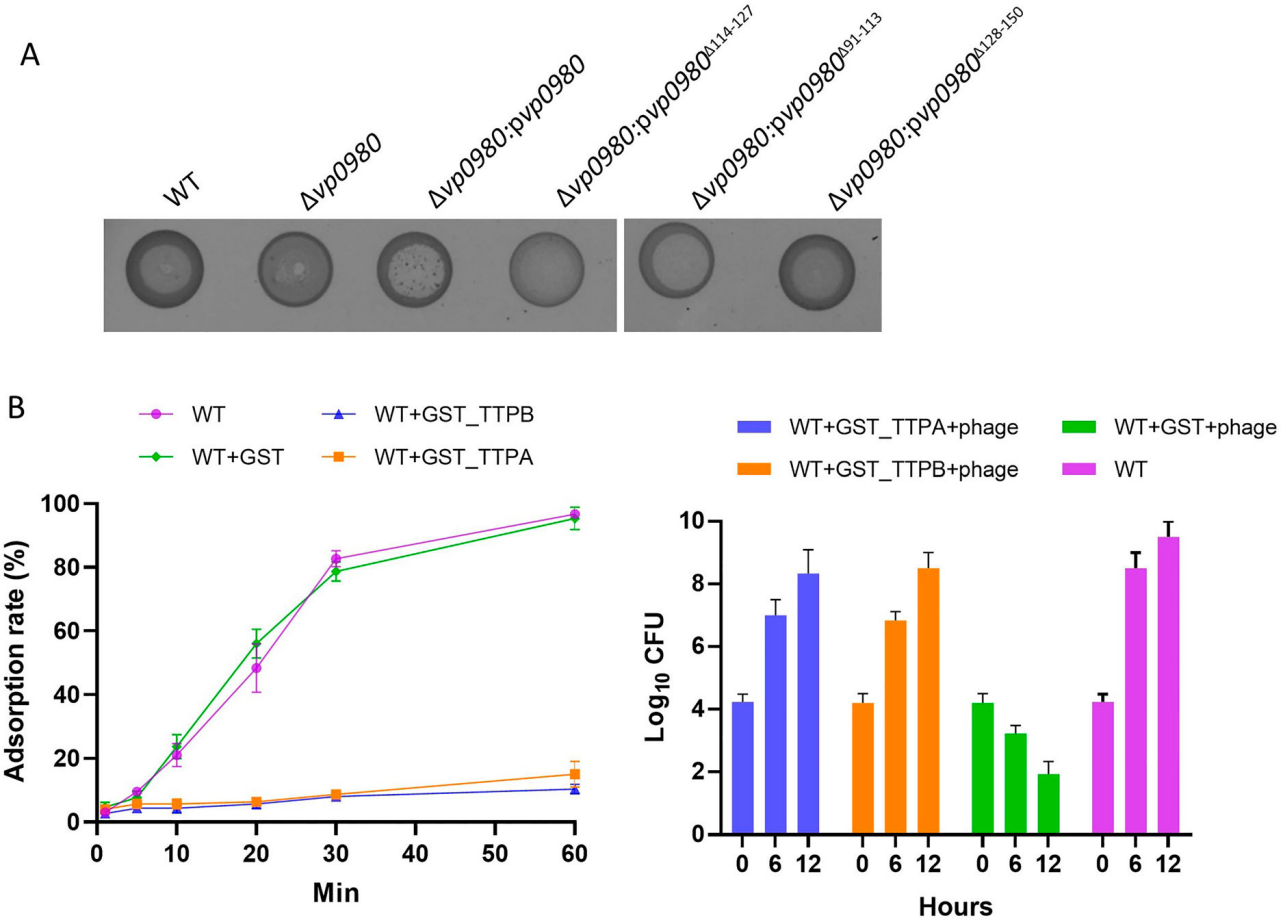
TTPA and TTPB binding with Vp0980 is essential for phage-mediated cell lysis. (A) A phage drop assay was performed to determine the role of different regions of Vp0980 in phage-mediated cell lysis. Each strain labeled at the top of the image was added with a drop of phage, and the lysis zone was visually observed. (B) (left) Adsorption quantification for the wild type or wild type in the presence of GST-TTPA, GST-TTPB or GST. B (right) The wild type was incubated with recombinant GST-TTPA, GST-TTPB, or GST for 1 hour. Subsequently, phage was added, and CFU were measured after 6 and 12 hours of incubation. The wild type without recombinant protein and phage was included as a control.

To determine whether binding of TTPA and TTPB with Vp0980 is important for phage adsorption and lysis of bacterial cells, we complemented the Vp0980 mutant with either WT *vp0980* (Δ*vp0980*:p*vp0980*) or *vp0980* lacking aa 114–127 (Δ*vp0980*:p*vp0980*^Δ114–127^) and performed phage adoption and phage drop assays. The results showed that the phage adsorption rate for Δ*vp0980*:p*vp0980*^Δ114–127^ was only ∼4%, which is comparable to that for Δ*vp0980* (Figure 2C), indicating that aa 114–127 are essential for phage adsorption. A phage drop assay showed that phage OWB could lyse Δ*vp0980*:p*vp0980* but not Δ*vp0980*:p*vp0980*^Δ114–127^ (Figure 6A), indicating that aa 114–127 are essential for phage-mediated bacterial cell lysis. In contrast, deletion of the transmembrane regions aa 91–113 or aa 128–150 had no effect on phage adsorption (Figure 2C) or phage-mediated bacterial lysis (Figure 6A), indicating that the exterior region but not the transmembrane region of Vp0980 is involved in phage adsorption. We further determined whether recombinant TTPA or TTPB could block the Vp0980-binding site and thus inhibit phage adsorption and phage-mediated cell lysis. The results showed that preincubation of wild type bacteria with GST-TTPA or GST-TTPB, but not GST, inhibited phage OWB adsorption (Figure 6B, left). Furthermore, phage OWB did not inhibit the growth of WT bacteria preincubated with recombinant GST-TTPA or GST-TTPB. In contrast, phage OWB inhibited the growth of WT bacteria preincubated with GST (Figure 6B, right). Taken together, these results demonstrated that Vp0980 binding with TTPA and TTPB is essential for phage adsorption and subsequent *V. parahaemolyticus* infection and cell lysis.

## Discussion

*V. parahaemolyticus* is a marine bacterial pathogen that can not only cause acute damage to the hepatopancreas organ of shrimp [44–46] but also cause gastroenteritis in humans [47–49]. Moreover, many *Vibrio* species are highly resistant to many commercially available antibiotics [50–53]. Therefore, alternative strategies to prevent and control *V. parahaemolyticus* infection are needed. Phage are ecofriendly antibacterial agents that are especially useful when alternative strategies to control pathogenic bacteria in aquaculture species are not available [54]. Multiple studies have been carried out using phage to control bacterial infections in shrimp, finfish, oysters, and Atlantic salmon [24,55,56]. However, bacteria can frequently become resistant to phage infection by interfering with phage interactions [57,58]. Thus, understanding the phage-bacteria interaction at the molecular level, particularly the phage ligand/bacterial receptor interaction, is crucial for the development of more effective phage therapies.

We previously isolated phage OWB from Atlantic Ocean water, which can effectively lyse the *V. parahaemolyticus* strain RIMD2210633 if the polar flagellum is deleted [26], suggesting that the polar flagellum inhibits phage infection. In this study, we showed that a naturally polar flagellum-deficient strain, ATCC17802, could be lysed by phage OWB, consistent with the conclusion that deficiency in the polar flagellum promotes phage OWB infection of *V. parahaemolyticus* [26]. To explore the molecular mechanisms of the phage-host interaction, we created a random transposon mutagenesis library with ATCC17802. A phage drop assay identified 8 mutants that were resistant to phage OWB infection (Figure 1B). Notably, the resistance of these 8 mutants to phage infection was not due to polar flagellum recovery, as these mutants were still defective in swimming (a key function of the polar flagellum). These mutated genes should be responsible for any steps during the phage infection process. For example, the cytoplasmic enzymes formyltetrahydrofolate deformylase, methylenetetrahydrofolate dehydrogenase, and dihydrofolate reductase are most likely involved in phage physiological processes, e.g. phage DNA replication, late gene expression, and particle packaging and assembly. Our results demonstrated that the catalytic site of Vp0879 (encoding a putative methylenetetrahydrofolate dehydrogenase) is required for phage-mediated cell lysis (Figure 1D) but not for adsorption (Figure 2C), indicating that the enzymatic activity of Vp0879 is involved in phage infection steps after adsorption. Studies have shown that some bacteriophages can produce dihydrofolate reductase for phage DNA ejection into bacterial cells [59–61]. Our results suggested that bacterial dihydrofolate reductase (Vp0333) is also required for phage infection (Figure 1B). The exact roles of these enzymes in phage infection need to be further explored. A LysR transcriptional regulator (Vp0635) has also been identified to be involved in the phage lytic process (Figure 1B). It is possible that Vp0635 regulates key bacterial events that are essential for phage replication, packaging or viral particle assembly. In this study, we identified only 8 ORFs that are involved in phage infection processes, probably because only ∼5,000 transposon mutagenesis mutants (1X coverage of the predicted ORFs in the genome) were screened. We were most interested in Vp0980, and the results demonstrated that Vp0980 is a receptor for phage adsorption and subsequent infection.

The most common structures used by tailed phages to recognize bacterial receptors are tail spikes, tail fibres and tail membrane-penetrating proteins [8]. Tail spikes mediate attachment to LPS and subsequently cleave the O-antigen to expose the outer membrane [62]. Tail fibres are structurally similar to tail spikes, but tail fibres are usually longer [63]. Tail fibres mediate adsorption by binding to receptors, including LPS, flagella, type 4 pili and outer membrane porins (ompC, ompF). Genome sequence analysis showed that phage OWB encodes four tail proteins: head–tail connector protein (OWB026), TTPA (OWB030), TTPB (OWB031) and tail fibre protein (OWB035). Our pulldown assay showed that the *V. parahaemolyticus* transmembrane protein Vp0980 specifically binds TTPA and TTPB but not tail fibre protein or the capsid proteins, indicating that Vp0980 is the receptor recognizing TTPA and TTPB. More importantly, Vp0980 lacking the outer membrane region was no longer able to bind TTPA and TTPB and could no longer mediate phage adsorption to *V. parahaemolyticus* cells, demonstrating that binding of the receptor Vp0980 with the phage ligands TTPA and TTPB is essential for phage adsorption. TTPA typically forms a ring below the tail tube and interacts with the phage fibres. TTPB forms the end of the tail below TTPA [15]. TTPA in *Klebsiella pneumoniae* bacteriophage KP32 has also been shown to have enzymatic activity to hydrolyze bacterial polysaccharides [42,43], but the role of TTPA in phage adsorption has not been experimentally shown. To the best of our knowledge, this study is the first time that tail structures TTPA and TTPB have been demonstrated to serve as ligands that recognize the conserved *Vibrio* receptor Vp0980 to mediate phage adsorption. Our results also showed that TTPA and TTPB do not bind tail fibres (OWB035) (Figure 4A). We reasoned that the tail fibre (OWB035) may bind other receptors, e.g. LPS, on *V. parahaemolyticus*. Our results demonstrated that the tail fibre binds LPS and mediates phage infection (Figure 5C and D). Thus, it is likely that adsorption of phage OWB to the host requires the interaction of not only the tail fibre with LPS but also the tail tubular proteins with Vp0980. TTPA and TTPB are present in not only all *Vibrio* phages but also phages that infect other bacterial species (e.g. *K. pneumoniae*). However, Vp0980 homologs are not observed in these bacterial species, and it is possible that Vp0980 functional orthologs are used as receptors for TTPA and TTPB to mediate phage adsorption to these bacterial species. It remains to be determined whether the binding of Vp0980 with TTPA and TTPB is responsible for reversible or irreversible adsorption.

In summary, we identified a conserved *Vibrio* transmembrane protein, Vp0980, that mediates phage adsorption by binding the phage ligand proteins TTPA and TTPB. Our findings highlighted the importance of this unprecedented receptor/ligand interaction in podophage infection of *Vibrio* species and possibly other bacterial species.

## Supplementary Material

Supplemental Material
