# Supplementary material for: Identification of a novel bacterial receptor that binds tail tubular proteins and mediates phage infection of Vibrio parahaemolyticus
Source: Emerg Microbes Infect. 2020 May 7;9(1):855–67. doi: 10.1080/22221751.2020.1754134 (PMC7241545; doi:10.1080/22221751.2020.1754134)
Supplement: Supplemental Material [file TEMI_A_1754134_SM0919.docx]

Table S1 Strains and plasmids used in this study

| Strains and plasmids | Relevant genotype/description | Source |
| --- | --- | --- |
| Strains |  |  |
| *V. parahaemolyticus* ATCC17802 | Wild-type | ATCC |
| Δ*vp0980* | ATCC17802 Δ*vp0980* | This work |
| Δ*LPS* | ATCC17802 Δ*vp0190-vp0214* | This work |
| Δ*vp0980*:p*vp0980* | Δ*vp0980* complemented with *vp0980* in pMMB207 | This work |
| Δ*vp0980*:p*vp0980*^Δ91-113^ | Δ*vp0980* complemented with *vp0980*Δ91-113 | This work |
| Δ*vp0980*:p*vp0980*^Δ114-127^ | Δ*vp0980* complemented with *vp0980*Δ114-128 | This work |
| Δ*vp0980*:p*vp0980*^Δ128-150^ | Δ*vp0980* complemented with *vp0980*Δ128-150 | This work |
| *E. coli* SM10λpir | KmR, *thi-1*, *thr*, *leu*, *tonA*, *lacY*, *supE*, *recA*::RP4-2-Tc::Mu, λpir | Laboratory stock |
| *E. coli* DH5αλpir | *supE44*, Δ*lacU169(ΦlacZΔM15)*, *recA1*, *endA1*, *hsdR17*, *thi-1*, *gyrA96*, *relA1*, λpir | Laboratory stock |
| Plasmids |  |  |
| pEVS104 | conjugative helper plasmid | [1] |
| pEVS170 | mini-Tn5-Em, *oriV*_R6Kγ__ *oriT*RP4 Kn^r^ | [1,2] |
| pVSV208 | Plasmid expressing a red fluorescent protein | [3] |
| pGEX-OWB027 | Sequences of OWB027 cloned into pGEX | This work |
| pGEX-OWB028 | Sequences of OWB028 cloned into pGEX | This work |
| pGEX-OWB030 | Sequences of OWB030 cloned into pGEX | This work |
| pGEX-OWB031 | Sequences of OWB031 cloned into pGEX | This work |
| pGEX-OWB035 | Sequences of OWB035 cloned into pGEX | This work |
| pMMB207-vp0980  pMMB207-vp0879 | vp0980 with 6xHis amino acid at the C-terminus inserted into pMMB207  vp0879 with 6xHis amino acid at the C-terminus inserted into pMMB207 | This work  This work |
| pMMB207-vp0980Δ91-113 | vp0980 lacking amino acids 91-113 cloned into pMMB207 | This work |
| pMMB207-vp0980Δ114-127 | vp0980 lacking amino acids 114-127 cloned into pMMB207 | This work |
| pMMB207-vp0980Δ128-150  pMMB207-vp0879K54A | vp0980 lacking amino acids 128-150 cloned into pMMB207  vp0879 with mutation of K54A cloned into pMMB207 | This work  This work |

Table S2 Primers used in this study

| Primer name | DNA sequence (5′-3′) |
| --- | --- |
| OWB027_FwBamH I | CGGGATCCATGCAAGATACGAATCTTCCACC |
| OWB027_ReEcoR I | CGGAATTCTTATCGCCCCAGTTGTTTGCC |
| OWB028_FwBamH I | CGGGATCCATGGCTGATACTTCATACAAAGCA |
| OWB028_ReEcoR I | CGGAATTCTTATGCTGGTTCTTCGAATTTAGTAA |
| OWB030_FwBamH I | CGGGATCCATGACCTTACTAGATGCAATCAATAT |
| OWB030_ReEcoR I | CGGAATTCCTATGTATATAACGCAGAGCGGA |
| OWB031_FwBamH I | CGGGATCCATGGCTCGACCATTTGAGGG |
| OWB031_ReEcoR I | CGGAATTCTTATACGCGACGACGGCGTT |
| OWB035_FwBamH I | CGGGATCCATGGCATTAAGTGTACAACGTGC |
| OWB035_ReEcoR I | CGGAATTCCTACTCTTGGTCTTTATCGTTAGAT |
| pmmbvp0980_1F | AGCTCGGTACCCGGGGATCCTCTAGTAAGGAGGTAGGATAATATGCCTAGAAAGCTGATAAAAC |
| pmmbvp0980_2R  pmmbvp0979_1F  pmmbvp0879_2R | TCTCATCCGCCAAAACAGCCAAGCTTTAATGGTGATGGTGATGATGTCTTACTTTTCGTTTTTGC  GGTACCCGGGGATCCTCTAGAAAAGTTATTCCTGTTGGTTATGACTGCTCAAAATATAGAC  TCTCATCCGCCAAAACAGCCAAGCTTTAATGGTGATGGTGATGATGTTGTTCAGTATGGAACTGC |
| pmmbvp0980_91_1R | CATGGTTATAGGGTTGCTGAC |
| pmmbvp0980_91_2F | GTCAGCAACCCTATAACCATGTTTGAGCTATCTTGGGAC |
| pmmbvp0980_114_1R | ATAGAATGGCTGAGGTGGAAC |
| pmmbvp0980_114_2F | GTTCCACCTCAGCCATTCTATATTGGGCCTCCATTCCTATTG |
| pmmbvp0980_128_1R | CGTGCTCATCTGTTGCATG |
| pmmbvp0980_128_2F  pmmbvp0879_K54A_1R  pmmbvp0879_K54A_2F | CATGCAACAGATGAGCACGATTCGCGGATTGTGGCGCTAC  TGCGCTGCCCACGTAAACTTG  CAAGTTTACGTGGGCAGCGCACGCCGCGCGTGTGAAGAAG |

Table S3 ORFs of the phage OWB genome and BLAST hits

| Phage OWB’s ORFs | BLASTP best match | Identity (%) |
| --- | --- | --- |
| Vp-OWB001 | hypothetical protein [VP93] | 100 |
| Vp-OWB002 | hypothetical protein [VP93] | 96 |
| Vp-OWB003 | hypothetical protein phiKDA1_10 [Enterobacter phage phiKDA1] | 59 |
| Vp-OWB004 | hypothetical protein VPP93_gp5 [VP93] | 94 |
| Vp-OWB005 | hypothetical protein VPP93_gp6 [VP93] | 86 |
| Vp-OWB006 | Putative peptidase [VP93] | 92 |
| Vp-OWB007 | putative primase [VP93] | 96 |
| Vp-OWB008 | putative DNA helicase [VP93] | 97 |
| Vp-OWB009 | hypothetical protein VPP93_gp12 [VP93] | 95 |
| Vp-OWB010 | putative DNA polymerase [VP93] | 96 |
| Vp-OWB011 | hypothetical protein VPP93_gp15 [VP93] | 66 |
| Vp-OWB012 | hypothetical protein VPP93_gp16 [VP93] | 84 |
| Vp-OWB013 | hypothetical protein VPP93_gp17 [VP93] | 94 |
| Vp-OWB014 | hypothetical protein VPP93_gp18 [VP93] | 94 |
| Vp-OWB015 | hypothetical protein VPP93_gp19 [VP93] | 84 |
| Vp-OWB016 | hypothetical protein VPP93_gp20 [VP93] | 94 |
| Vp-OWB017 | hypothetical protein VPP93_gp21 [VP93] | 98 |
| Vp-OWB018 | putative exonuclease [VP93] | 99 |
| Vp-OWB019 | MULTISPECIES: hypothetical protein [Streptomyces] | 64 |
| Vp-OWB020 | putative endonuclease [VP93] | 99 |
| Vp-OWB021 | hypothetical protein VPP93_gp24 [VP93] | 98 |
| Vp-OWB022 | / | / |
| Vp-OWB023 | putative RNA polymerase [VP93] | 98 |
| Vp-OWB024 | / | / |
| Vp-OWB025 | hypothetical protein VPP93_gp26 [VP93] | 98 |
| Vp-OWB026 | putative head-tail connector protein [VP93] | 98 |
| Vp-OWB027 | putative scaffolding protein [VP93] | 94 |
| Vp-OWB028 | putative capsid protein [VP93] | 96 |
| Vp-OWB029 | / | / |
| Vp-OWB030 | putative tail tubular protein A [VP93] | 97 |
| Vp-OWB031 | putative tail tubular protein B [VP93] | 96 |
| Vp-OWB032 | hypothetical protein VPP93_gp32 [VP93] | 91 |
| Vp-OWB033 | hypothetical protein VPP93_gp33 [VP93] | 93 |
| Vp-OWB034 | putative internal core protein [VP93] | 98 |
| Vp-OWB035 | putative tail fiber protein [VP93] | 94 |
| Vp-OWB036 | putative glycosyl hydrolase [VP93] | 98 |
| Vp-OWB037 | putative DNA maturase A [VP93] | 97 |
| Vp-OWB038 | putative DNA maturase B [VP93] | 98 |
| Vp-OWB039 | hypothetical protein VPP93_gp39 [VP93] | 97 |
| Vp-OWB040 | hypothetical protein VPP93_gp40 [VP93] | 37 |
| Vp-OWB041 | / | / |
| Vp-OWB042 | hypothetical protein VPP93_gp41 [VP93] | 93 |
| Vp-OWB043 | hypothetical protein VPP93_gp42 [VP93] | 99 |

1. Adin DM, Visick KL, Stabb EV. Identification of a cellobiose utilization gene cluster with cryptic beta-galactosidase activity in Vibrio fischeri. Appl Environ Microbiol. 2008 Jul;74(13):4059-69.

2. Lyell NL, Dunn AK, Bose JL, et al. Effective mutagenesis of Vibrio fischeri by using hyperactive mini-Tn5 derivatives. Appl Environ Microbiol. 2008 Nov;74(22):7059-63.

3. Dunn AK, Millikan DS, Adin DM, et al. New rfp- and pES213-derived tools for analyzing symbiotic Vibrio fischeri reveal patterns of infection and lux expression in situ. Appl Environ Microbiol. 2006 Jan;72(1):802-10.
